# Dietary Approaches for Complementary Feeding: The Greek Mediterranean Diet as a Model for Caregivers

**DOI:** 10.3390/children11111310

**Published:** 2024-10-29

**Authors:** Sofia Eleftheriou, Emilia Vassilopoulou, Anastasia Barbouni, Michael Chourdakis, Anastasia Kanellou

**Affiliations:** 1 Department of Food Science and Technology, Faculty of Food Science, University of West Attica, Egaleo,12243 Athens, Greece; akanellou@uniwa.gr; 2 Department of Nutritional Sciences and Dietetics, School of Health Sciences, International Hellenic University, 57400 Thessaloniki, Greece; vassilopoulouemilia@ihu.gr; 3 Department of Clinical Sciences and Community Health, Univertià degli Studi die Milano, 20122 Milan, Italy; 4 Department of Public and Community Health, School of Public Health, University of West Attica, Egaleo, 12243 Athens, Greece; abarbouni@uniwa.gr; 5 School of Medicine, Faculty of Health Sciences, Aristotle University of Thessaloniki, 54124 Thessaloniki, Greece; mhourd@gapps.auth.gr

**Keywords:** complementary feeding, Mediterranean diet, practical guidance, Greek diet, infant nutrition

## Abstract

Background/Objectives: Complementary feeding (CF), is defined as the process of providing foods in addition to milk when breast milk or milk formula alone are no longer adequate to meet nutritional requirements. CF affects not only growth but also the subsequent development of the child’s dietary preferences. The Mediterranean diet (MedDiet) is one of the most studied dietary patterns worldwide. The main purpose of this article is to provide practical guidance for CF to caregivers introducing the Greek MedDiet, summarizing the current different approaches. The evidence-based information provided could be used by developers to create a reliable digital app for CF based on Mediterranean foods and traditional dishes. Methods: An in-depth literature review of the existing guidelines and recently published research data on CF using PubMed, national recommendations, and grey literature were undertaken. Results: Practical, simple, evidence-based, comprehensive tables are presented. The tables serve as a guide to simplify the process of introducing Mediterranean foods in CF. Τhe tables consist of a total of more than 90 foods and day-to-day guidance for the first 13 weeks of CF. Furthermore, specific dietary guidance and suggestions regarding the order and content of meals during CF are given. Discussion: The tables are based on updated recommendations, summarizing the optimal feeding practices in a MedDiet-based perspective. Conclusions: Conclusively, there are significant contradictions among different organizations that present practical issues and cause confusion among caregivers. An effort was carried out to provide practical evidence-based guidance for caregivers introducing MedDiet during CF.

## 1. Introduction

Complementary feeding (CF), as defined by the World Health Organization (WHO), is the process of introducing foods in addition to milk when breast milk or formula alone are no longer sufficient to meet nutritional requirements [1]. As CF begins, children enter a period of growth and development, marked by significant changes due to exposure to new foods, tastes, and feeding experiences. This period is crucial for establishing healthy habits and facilitates the acceptance of healthy foods and beverages, laying the foundation for long-term dietary patterns [2]. However, there is considerable variation in CF recommendations and practices both between and within countries. The timing, meal composition, and methods of CF differ significantly across regions due to cultural and socioeconomic factors, often leading to confusion among caregivers. Since the CF process impacts the health, development, and behavior of young children, it is crucial to provide practical guidance for caregivers [3].

The Mediterranean diet (MedDiet) [4], one of the most studied and well-known dietary patterns worldwide, has been associated with numerous health benefits. In 2013, the United Nations Educational, Scientific and Cultural Organization (UNESCO) acknowledged the MedDiet as an Intangible Cultural Heritage of Humanity [5] both for its important health and nutritional outcomes and for its environmental impact. Regarding the MedDiet’s health benefits, it is recognized as a healthy eating pattern for the prevention of cardiovascular diseases, promoting longevity and supporting healthy aging. People living in Greece and other people living around the Mediterranean Sea, however, have ceased embracing the MedDiet [6,7,8]. Moreover, infants and young children worldwide consume increasing amounts of junk and unhealthy foods that are full of sugars, fats, salt, and refined carbohydrates. Beyond the adverse health outcomes associated with the content of these foods, unhealthy foods also displace the intake of healthy foods that contain essential vitamins and minerals [9]. Many of the commercial foods specially formulated for young children also contain high amounts of sodium and sugar. As eating habits are built from a very young age, it is a challenge to create the conditions during the CF period to adopt more of the Mediterranean way of life [2,10,11].

Although numerous digital applications (apps) address infant feeding and Baby-Led Weaning (BLW) methods, to our knowledge, none specifically focus on CF and the MedDiet. Furthermore, the quality of information provided by smartphone apps is often poor [12]. Evidence-based apps that align with National Health Infant Feeding Guidelines and are developed by healthcare professionals can ensure that parents have access to credible and reliable resources [13].

The main purpose of this article is to offer practical guidance for caregivers on introducing CF based on the Greek MedDiet [14] during the first months of CF. Additionally, this article aims to review the current recommendations, provide practical advice, and introduce tables that may serve as a guide for developing an evidence-based digital app for CF, featuring Mediterranean foods and traditional dishes. Since digital dietary applications have been shown to be highly effective in promoting health behaviors and providing personalized nutritional recommendations [15], the development of such an app would be a valuable tool for caregivers.

This article focuses on practical dietary guidance for CF based on a healthy dietary pattern [14], the Greek MedDiet, in healthy, term-born infants and does not address the needs of children with (or recovering from) acute malnutrition and serious illness, children living in emergencies, or children who are disabled or at risk of iron depletion, including exclusively breastfed infants by mothers with low-iron status, early umbilical cord clamping, preterm birth, small for gestational age, or those with high growth velocity.

Considering different aspects of CF, the discussion addresses the recent data provided concerning proper timing for starting CF and introducing allergens, methods of feeding, methods of adding new foods, the order of new foods added, foods and beverages to be avoided, proper type of diets, and the significance of diversity.

## 2. Materials and Methods

A literature review of the existing guidelines and recently published research data on CF using PubMed, National Recommendations, and grey literature was conducted. Specifically, the following 5 steps were adopted:

Step 1: We formulated the research question to identify the best feeding practices for CF and the available digital applications for CF. The objective was to offer practical guidance for caregivers on introducing CF based on the Greek MedDiet.

Step 2: We conducted a literature review using PubMed. The keywords that were used are as follows: “complementary feeding”, “solid foods guidelines”, and “food introduction for infants”.

Step 3: We searched the guidelines from official sites of related organizations worldwide, such as the World Health Organization, American Academy of Pediatrics, Center of Disease Control, European Society for Pediatric Gastroenterology, Hepatology, and Nutrition, Australasian Society of Clinical Immunology and Allergy, and European Food Safety Authority, and published national recommendations, like those from the National Health and Medical Research Council of Australia, Greek Ministry of Health, and National Health System of Great Britain.

Step 4: A survey of the available digital apps for CF was conducted by reviewing Play Store and App Store. The keywords that were used are as follows: “complementary feeding”, “solid foods guidelines”, and “food introduction for infants”.

Step 5: We constructed a practical, easy-to-follow, evidence-based comprehensive table aimed at serving as a guide for caregivers to simplify the process of introducing new foods in CF.

## 3. Results

Practical guidance for caregivers is summarized in the tables provided. Table 1, Table 2 and Table 3 present integrated applicable guidance for caregivers as an effort to summarize current practices for CF in a MedDiet-based perspective. The selection of foods in Table 2 and Table 3 is not mandatory. The order, the type, and the frequency of the foods added should be adjusted according to seasonal availability, family preferences, child’s developmental readiness, and individual needs. Additionally, the tables offer a comprehensive overview of data that could be used as a base to develop an evidence-based app for CF, including Mediterranean foods and traditional dishes. These tables are consistent with the updated guidelines and recent data provided [16,17,18]. Specifically, Table 2 serves as an indicative practical example on introducing solid foods during CF according to a Greek MedDiet that reminds caregivers to add the most popular allergenic foods as soon as possible. The tables summarize evidence-based recommendations by the WHO [1], Greek National Health Infant Feeding Guidelines [19], and other reliable sources. Specifically, Table 2 presents a day-by-day CF diet model for the first 13 weeks of CF, summarizing all recent data into a practical guide. For the first 3 days, it is recommended to replace one milk feed with fruits and, from the 4th day, a second milk feed with vegetables. For the main meal, it is recommended to offer the same dish for 3 consecutive days in order to give time to the infants to get used to the new tastes, but it also advisable to gradually increase the portion sizes. As the baby gets used to the weaning, a gradual increase in the number of meals provided is recommended.

### 3.1. Practical Guidance for Caregivers Introducing MedDiet

Table 1 summarizes the basic principles of CF regarding introducing the MedDiet.

Table 3 summarizes the majority of foods introduced in Table 2, which are organized by their respective food groups, with common allergens highlighted in bold [20].

### 3.2. Criteria for the Selection of Foods and Definition of the Order Provided

The MedDiet foods and traditional Greek dishes in Table 2 and Table 3 have been selected [21] in order to introduce CF guidelines in a MedDiet perspective [21] to ease the transition towards a Mediterranean feeding model of eating [10,11,22]. The foods that have been selected are foods that the MedDiet is characterized by, like seasonal vegetables and fruits, unrefined cereals, nuts, legumes, seeds, and extra virgin olive oil as the primary source of fat. The health benefits attributed to extra virgin olive oil are due to its high nutritional quality and multiple positive effects on health [23]. Additionally, seafood, dairy products, meat, eggs, and fermented products, like ariani and traditional cheeses, have also been included. Specifically, the selection of foods in Table 2 and Table 3 are based on seven parameters: digestibility (those more digestible are more preferable for starting), nutrition density (the more nutrients, the better it is) [24], availability in Greek region, texture (softer textures are more easily included at first) [25], allergenicity (earlier introduction of multiple allergenic foods may minimize an infant’s risk for developing a food allergy) [26], seasonality, and sustainability [27]. As far as the order of fruits is concerned, seasonal soft-textured options, such as pear in the winter and peach in the summer, are recommended to start with. For example, pear is being selected first due to its soft, buttery texture when ripe, making it easy to mash with a fork. In the summer, a fine selection of seasonal fruits (melons and watermelon) is recommended. It is important to introduce ripe fruits peeled and without seeds for better digestion. Caregivers can offer fruits separately or mix every new fruit together with others that are well tolerated according to the following method: It is recommended to start with a very small portion (1/8 teaspoon) of every new food added. When adopting the conventional method of blended foods, it is recommended that this practice should be replaced soon by chopped or fork-mashed foods and then be replaced by small pieces that the child can consume with hands until the 10th month of age. Blended foods beyond the 10th month of age are not recommended [19].

## 4. Discussion

The updated guidelines on CF have been summarized and discussed in this section to identify optimal feeding practices. The data presented in Table 1, Table 2 and Table 3 is based on recent recommendations for CF, combining elements from the Greek MedDiet. The proportion of main nutrients recommended during CF are in line with the MedDiet [28], since fat should constitute 40% of the energy intake, and proteins should be 15% of the energy intake from 6 to 12 months [29]. To help infants become accustomed to the Mediterranean way of life during the CF period [2], Table 2 and Table 3 include foods and traditional MedDiet dishes [21]. Since MedDiet dishes contain a substantial amount of vegetables and dietary fiber, with their energy content mainly derived from olive oil [30], we recommend adopting traditional recipes and dishes according to the recommendations for CF to ensure easy digestibility, avoiding added salt or ingredients like hot spices. We recommend preferably not using olive oil during cooking but adding fresh extra virgin olive oil after cooking. Blended foods are not recommended beyond the 10th month of age [19], making it challenging for caregivers to introduce MedDiet dishes and similar types of foods as consumed by the rest of the family as early as possible. The goal is to transition the infant gradually to consuming the same foods as the rest of the family by 12 months of age. We offer guidance for the first 13 weeks of CF, considering this period critical for establishing and covering the variety of Greek MedDiet dishes. Regarding the age of CF introduction, we do not provide specific recommendations, as this depends on the infant’s growth and developmental status. We propose starting either with the traditional spoon-fed weaning method or other methods, depending on the child’s preferences and the caregiver’s readiness. The early introduction of allergens and a wide variety of foods is encouraged, which is why we suggest adding one new food every day, following practices from Great Britain [17], Canada [17,31], and Australia [16,32]. However, this is merely a suggestion; caregivers may choose to introduce new foods at intervals of 2 to 7 days. Considering different aspects of CF, we summarize recent data on key factors: the appropriate timing for CF initiation, allergen introduction, feeding methods, time intervals between introducing new foods, the order of new foods added, foods and beverages to avoid, proper types of diets, and the importance of diet diversity.

### 4.1. Summary and Discussion of CF Recommendations

#### 4.1.1. Age of CF Initiation

According to WHO recommendations, infants should start receiving complementary foods at 6 months of age in addition to breast milk or infant formula [1]. However, it is pointed out that the recommendation regarding the age of introduction of CF after 6 months is a public health recommendation and recognized that some infants may benefit from earlier introduction of complementary foods. Also, the American Academy of Pediatrics, in the most recent edition of the Pediatric Nutrition Handbook, recommends human milk as the sole food item for healthy, term infants for about the first 6 months of life and the introduction of complementary foods at approximately 6 months [33]. However, in 2019, the European Food Safety Authority and Novel Foods and Food Allergens (EFSA and NDA) stated that for infants who exhibit the developmental skills needed to consume foods, solids could be introduced before the age of 6 months [34]. Earlier in 2017, the European Society for Pediatric Gastroenterology, Hepatology, and Nutrition (ESPGHAN) Committee on Nutrition had recommended that complementary foods should not be introduced before the age of 4 months but not delayed beyond the age of 6 months [29]. The Committee further noted that there are practical problems with guidelines that cause confusion among caregivers. In line with this, the Healthy Eating Research Project, which focused on promoting healthy growth in young children in the United States, recommended exclusive breastfeeding for at least 4 months and the introduction of complementary foods between the ages of 4 to 6 months [35]. The publication does not explain the apparent contradiction, at least for breastfed infants, of different recommended timings. In Greece, recommendations for the introduction of solid foods cited by the Greek Ministry of Health support the WHO recommendations that suggest CF after 6 months [19]. As such, the discrepancies among guidelines from different organizations could be explained by the nutritional adequacy of breast milk for breastfeeding infants, growth outcomes, health outcomes, and developmental readiness [36]. It is apparent that the proper time for starting CF is determined according to infant’s growth and developmental status.

#### 4.1.2. Traditional vs. Modern Methods of CF

According to WHO recommendations, infants should initially receive complementary foods 2–3 times a day between 6 and 8 months and increase to 3–4 times daily between 9 and 11 months and 12 and 24 months. Additional nutritious snacks should also be offered 1–2 times per day for the ages of 12–24 months, as desired. It is recommended to increase food consistency and variety as the infant gets older, adapting to the infant’s requirements and abilities. Infants can eat pureed, mashed, and semi-solid foods beginning at 6 months. By 8 months, most infants can handle “finger foods”. By 12 months, most children can eat the same types of foods as consumed by the rest of the family while keeping in mind the need for nutrient-dense foods, including animal-sourced foods like meat, poultry, fish, eggs, and dairy products [1]. In recent years, however, there is growing interest in alternative weaning methods; specifically, a new approach to introducing solid foods, Baby-Led Weaning (BLW), has been developed [37]. This method is an alternative to traditional feeding practices, where the parent typically feeds the infant mashed food using a spoon. Instead, BLW encourages infants, starting at 6 months of age, to self-feed, promoting their active participation and psychomotor skill development. The goal is to gradually offer the child food in pieces that they can manage independently, although all feedings should still be supervised by an adult. BLW may offer various benefits, including improved autonomy, better appetite control, and greater satiety responsiveness, leading to healthier eating patterns. However, recent studies indicate that BLW is associated with a higher incidence of gagging, spitting out food, and triggering the vomiting reflex compared to traditional spoon feeding. Despite this, no significant differences in choking incidents, body mass indexes (BMIs), or overall energy intakes have been observed between infants using the BLW method and those fed traditionally [38]. The impact of BLW on a child’s diet is heavily influenced by their family’s food choices. If the family’s diet is not nutritionally adequate, the child may be at increased risk of consuming excessive sodium, saturated fat, and protein. Although animal protein is essential for growth, excessive intake may negatively affect a child’s health [39]. In families following a vegetarian/vegan diet, careful attention must be paid to ensure vitamin B12, vitamin D, iron, calcium, zinc, folic acid, protein, and omega-3 fatty acid intakes are adequate. Many pediatricians are cautious about vegan/vegetarian weaning methods, and their hesitance may lead parents to begin weaning without medical supervision, increasing the risk of severe nutritional deficiencies in the child [40]. A modified approach, “Baby-Led Introduction to Solids” (BLISS), has been developed to address potential concerns about iron status, choking, and growth faltering. BLISS has been shown to increase the range of iron-rich foods consumed, reduce food fussiness, and has resulted in infants exhibiting greater enjoyment of food compared to those who are spoon fed. However, BLISS has not demonstrated a significant advantage in promoting a more appropriate body weight than traditional feeding method [41,42].

#### 4.1.3. Proper Timing of Allergen Introduction

For many years, the American Academy of Pediatrics and allergy associations discouraged the introduction of allergenic foods, such as peanuts, eggs, and shellfish, until 1 year old or older to prevent allergenic reactions. However, this advice was not universally adopted, as no controlled studies at the time confirmed the risk of early allergenic food introduction. In 2019, the American Academy of Pediatrics updated its stance, noting that delaying the introduction of allergenic foods, beyond 4 to 6 months, including peanuts [26], eggs, and fish, does not prevent atopic disease [43]. The current consensus among pediatric and allergy associations is that delaying allergenic food introduction beyond 12 months is unnecessary [29,43]. Evidence suggests that introducing common allergenic foods within the first year of life may reduce the risk of developing food allergies. The ESPGHAN Committee on Nutrition recommends that allergenic foods can be introduced as soon as CF begins (which can occur any time after 4 months of age), a view supported by the EFSA and NDA Panel, which also endorses the early introduction of gluten alongside other foods [34]. This recommendation contrasts with the WHO, which recommends CF after 6 months [1]. ESPGHAN further recommends offering a variety of flavors and textures, including bitter-tasting vegetables. Whole cow’s milk, however, should not be the main drink before 12 months of age. For infants at a high risk of peanut allergy—those with severe eczema, an egg allergy, or both—peanut introduction is advised between 4 and 11 months following an evaluation by an appropriately trained specialist. Gluten can be introduced between 4 and 7 months, but large quantities should be avoided during the first weeks of its introduction and later during infancy. All infants should receive iron-rich complementary foods, including meat products and/or iron-fortified foods. The addition of sugar or salt to complementary foods should be avoided, as should fruit juices or sugar-sweetened beverages. Vegan diets should only be followed under appropriate medical or dietetic supervision, as failure to follow supplementation guidelines can lead to serious nutritional deficiencies. Recent data support the earlier introduction of allergens. A systematic review and meta-analysis found that introducing multiple allergenic foods within the first year of life is associated with a lower risk of developing food allergies. These findings support the concept of introducing allergenic foods before 6 months to prevent food allergies, contrary to the WHO’s infant feeding guidance [44]. The Greek Ministry of Health recommendations support that the order of added foods and the selection of foods added for the first time in the diet of the infant are not strictly defined and differ between most regions depending on their dietary tradition and food availability. Additionally, the Greek Ministry of Health recommends that CF starts with cereals, vegetables, fruits, and meat. The introduction of the most common foods, since they are well tolerated by the child, is followed by the most potent allergenic foods, such as cow’s milk protein (yogurt and cheese but not cow’s milk), eggs, soy, wheat, fish and seafood, peanuts, and all nuts. The administration of cow’s milk is recommended after the 12th month [19]. Conclusively, it is recommended that common allergens (such as peanuts, eggs, wheat, and cow’s milk) should be regularly included in an infant’s diet during the first year of life [32]. This is consistent with the WHO recommendations encouraging food diversity in the weaning diet.

#### 4.1.4. Methods of Introducing New Foods

The prevailing guideline in the current literature is that each new food should be introduced gradually into the infant’s diet and added one at a time at 2–7 day intervals. Food combinations can be given to older infants after their tolerance to the individual components or foods is proved, to avoid possible allergic reactions [45]. The American Academy of Pediatrics [46] and the Centers for Disease Control and Prevention (CDC) [18] recommend introducing one single-ingredient food at a time and observing the infant for 3 to 5 days between the introduction of each new food to monitor for allergic reactions. The Greek Ministry recommendations for CF [19] point out waiting 2–7 days between introducing new foods. On the contrary, the Australian Government Department of Health does not recommend gradual introduction and a waiting period between new added foods [8]. The recommendations of the National Health System (NHS) of Great Britain do not suggest such a restriction either [17]. The NHS’s guidelines for CF address only that foods containing allergens (such as peanuts, hens’ eggs, gluten, and fish) can be introduced one at a time and in small amounts so that any reaction can be spotted. The Australasian Society of Clinical Immunology and Allergy (ASCIA) [16] pointed out the following: “only introduce one common allergy causing food at each meal, so that the problem food can be easily identified if there is an allergic reaction”. Furthermore, the ASCIA focuses on the fact that if a baby has no allergic reaction to the common allergy causing foods, it is important to include these foods in the baby’s meals about twice a week. A joint statement from Health Canada, the Canadian Pediatric Society, Dietitians of Canada, and Breastfeeding Committee for Canada does not mention anything about intervals between new added foods [31]. Yet with advances in the understanding of infant food diversity, the guidance that pediatric practitioners are providing to parents is unclear [44]. A recent study revealed that most pediatric practitioners did not advise waiting 3 days or more between introducing new foods unless the infant was at risk for food allergies, suggesting that the current recommendation may limit food diversity and delay early common allergen introduction. This shift indicates a need to re-evaluate the feeding guidelines [47]. In Greece, a survey of pediatricians found discrepancies in CF practices despite evidence-based guidelines. Pediatricians often delay introducing common allergens and recommend longer intervals between new foods, particularly for high-risk infants [3]. Conclusively, there is no evidence-based determined interval between different foods added, although longer intervals may lead to a delay in introducing common allergens.

#### 4.1.5. Diversity, Diet Types, and the Significance of Responsive Feeding

A lack of dietary diversity increases the risk of micronutrient deficiencies, but its importance extends beyond meeting nutrient needs. Diverse diets expose children to different tastes and textures, creating nutrient synergies that aid absorption. Increasingly, infants are consuming unhealthy foods that are high in sugars, fats, salt, and refined carbohydrates, which displace nutrient-dense foods. Many commercial baby foods also contain excessive sodium and sugar. Avoiding entire food groups is generally not advised. Vegan diets should only be followed under medical or dietetic supervision, as failure to supplement correctly can lead to serious deficiencies [29]. Current evidence suggests that even vegetarian diets during CF carry a high risk of critical micronutrient deficiencies, potentially leading to growth retardation and developmental delays compared to a healthy omnivorous diet, such as the MedDiet. During CF, a big variety of foods that are nutrient-dense is recommended [48]. Preferably, these foods should come from local sources and be fresh and rich in essential micronutrients. There are three food groups that are essential for optimal growth: Fruits and vegetables (FV); nuts, pulses, and seeds (NPS); and animal-sourced foods (ASF) [1]. The WHO recommends consuming FV daily, as FV are a good source of many vitamins and minerals. NPS is a particularly important food group, as these can add nutritional value to diets when ASF are not accessible. FV and NPS comprise a basic component of the Greek MedDiet [14]. According to WHO’s guidelines, ASF (meat, poultry, fish, or eggs) should be eaten daily, or as often as possible, since they are considered more nutritionally dense, with high sources of energy and readily digested protein [1].

Responsive feeding (RF) is defined as “feeding practices that encourage children eat autonomously and in response to physiological and developmental needs, which may encourage self-regulation in eating and support cognitive, emotional and social development” [1]. RF can help reduce the risk of undernutrition and obesity by teaching children to self-regulate food intake. Family meals and social events offer opportunities for children to observe, imitate, and develop healthy eating habits. Therefore, the way that a child is being fed is as important as the quality of food that a child is being fed [9,49]. Conclusively, a balanced omnivorous diet like the Greek MedDiet, providing a wide range of fresh and cooked foods, combined with RF practices seems to remain the best option during CF. Table 4 summarizes RF recommendations for caregivers.

#### 4.1.6. Order of Adding New Foods, Textures, and Processing

There is no specific order to add new foods [19,35]. Ideally, the introduction of solid foods should be done while the baby continues to breastfeed. According to Greek National Health Infant Feeding Guidelines, cereals, vegetables, and fruits are a good choice for the first-introduced solid foods. Then, it is recommended to add good sources of animal protein and iron-rich products, such as red and white meat. Since these foods are well tolerated by an infant, then, egg and fish can be introduced. By the end of the first year of life, it is recommended that a big variety of foods from all food groups are introduced into the infant’s diet. Almost all well-cooked vegetables can be eaten by an infant. Firstly, it is recommended to choose seasonable fresh vegetables and fruits that would be cooked until they are soft. Fruit can be eaten after peeling and crushing them using a fork if they are soft enough (e.g., apricot, banana, melon, peach, and pear) or after they are blended if they are hard (e.g., apple and pear). Drinking fresh fruit juices is not recommended in infancy, as well as commercial fruit juices or compotes, as they contain added sugars or preservatives. It is recommended that since the number of foods a baby can eat increases, a variety of fruits and vegetables would be the best choice for infants. The introduction of red meat and white meat, due to their content of proteins of high biological value, iron, and zinc, which are components necessary for the development of the infant, should not be delayed and should be introduced until the end of the 7th month of life. Fish and eggs can be introduced gradually and in small quantities before the first year of life [29]. Regarding other grains that contain gluten, such as wheat, barley, and rye, it is recommended to add these while the child is still breastfeeding and until the end of the 7th month [29]. These choices are being followed by bread, pasta, and other cereals. Legumes are recommended to be added into the infant’s diet towards the end of the first year of life after all the above food groups have been well tolerated. During this period of CF, milk is still the basis of an infant’s diet. In any case, breastfeeding is recommended during this period. If a baby does not breastfeed, (s)he should consume infant formula, which is suitable for the second half of life. Cheese and yogurt can be gradually included in small amounts in the infant’s diet after the above foods are well tolerated. Fresh unadulterated cow’s milk is recommended to be introduced into the diet of infants after the 12th month [19]. The same applies to fresh goat’s milk or the milk of other animals. Simultaneously, infants need drinking water during this period of CF. According to the CDC [18], “By the time children are 7 or 8 months old, they can eat a variety of foods from different food groups. These foods include infant cereals, meat or other proteins, fruits, vegetables, grains, yogurts and cheeses, and more”. The proper time for adding new foods is summarized in Table 5.

#### 4.1.7. Foods and Beverages to Be Avoided or Limited

Based on current scientific guidelines, it is recommended that the consumption of fresh milk, added sugars, and low-calorie sweeteners, as well as foods with high sodium content and unpasteurized foods and beverages should be avoided at least until the first year of age. Processed meat and its products should be avoided as well due to their high contents of salt, fat, and preservatives. Foods in forms that may cause choking, such as hard pieces of fruit, whole grapes, whole cherry tomatoes, fruit with stones like whole cherries, raw carrots, whole nuts, and fish with bones should be avoided entirely. Additionally, it is recommended to avoid drinks with low nutrient value, such as tea, coffee, and sugary soft drinks and limit the amount of juice offered to avoid displacing more nutrient-rich foods. [1]. Recently, the American Academy of Pediatrics (AAP) recommends avoiding rice products, such as rice milk, rice cakes, rice biscuits, rice cereals, and brown rice syrup during CF, since rice tends to absorb more arsenic from groundwater than other crops [51]. Other options instead of rice cereal include oat, barley, and multigrain cereal. The EFSA points out that this type of foodstuff indicated for the young population made a relevant contribution in dietary exposure to arsenic in this age group [52]. Concerns have also been raised about arsenic in apple juice and other juice products as well. During infancy and up to the age of 12 months, honey should not be consumed by infants as it may contain Clostridium Botulinum spores, which is the causative agent of botulism [1]. Table 6 summarizes foods and beverages to be avoided or limited to 12 months of age.

## 5. Conclusions

A balanced omnivorous diet, like the MedDiet, combined with RF practices seems to remain the best option during CF. To improve the nutritional status, as well as future dietary preferences of children, the nutritional education provided to caregivers is of great importance. Successful strategies according to CF depend on accurate information and a supportive environment from family, community, and the healthcare system. This article provides practical guidance for introducing CF based on the Greek MedDiet perspective, involving RF, during the first weeks of CF. The tables serve as a flexible and easy-to-follow guide contributing to creating feeding plans tailored to seasonality, availability, developmental readiness, and individual needs. Furthermore, the tables support the development of a user-friendly and evidence-based digital app for CF based on Mediterranean foods and traditional Greek dishes. Since there are only few studies that investigated the role of the MedDiet during CF [53] until now, further research should be conducted in order to assess the effect of the MedDiet, such as the Greek MedDiet model provided, in future diet preferences of children and health outcomes.

## Figures and Tables

**Table 1:** Practical principles for caregivers introducing the MedDiet [19].

Start with one meal a day and gradually add a second and third as the child grows up during their 1st year of life.
Keep a diary of new foods introduced and any reactions from eating them.
Start with more easily digested fruits and vegetables, especially peeled fruits and boiled vegetables, and gradually increase the quantity and quality of the selected foods. First, choose to introduce more digestible foods and preferably foods in their most digestible form, e.g., legumes that are easier to digest, such as red lentils and shelled chickpeas.
Observe for any unusual reactions, such as skin rash, diarrhea, or vomiting. If you notice any of these symptoms, stop feeding the new food and tell your pediatrician about the reaction as soon as possible.
Insist on the exposure of each new food so that your infant gradually accepts it. It may take eight to fifteen exposures of the food before it is accepted.
If the baby has no allergic reactions to the common allergens, it is important to include these allergenic foods in the baby’s meals about twice a week.
It is recommended to avoid adding salt, simple sugars, and hot spices. One teaspoon of extra virgin olive oil should be added after cooking for the main meals.

**Table 2:** CF day-by-day diet model based on Greek MedDiet.

Weeks	Days	New Food	1st Meal (Before Lunch or Before Dinner)		2nd Meal (Lunch and/or Dinner)		Extra Meal
			Food	Quantity	Food	Quantity	
1st	1st	**Peeled pear**	Pear	¼–½ pear or 1–2 tsp.	-		-
	2nd	**Blended apple**	Fruits	¼–½ pear, ¼–½ apple, or 1–2 tsp.			-
	3rd	**Mashed banana**	Fruits	¼–½ pear, ¼–½ apple, and ¼ banana			-
	4th	**Vegetable soup**	Fruits	¼–½ pear, ¼–½ apple, and ¼ banana	Carrot, potato, zucchini, celery, and lemon	Gradual introduction (1–2 tsp. first day, 2–4 tsp. second day, etc.)	-
	5th	**Peeled avocado**	Fruits	¼–½ pear, ¼–½ apple, and ¼ avocado			-
	6th	**Melon**	Fruits	1–2 Tbsp.			-
	7th	**Pumpkin**	Fruits	1–2 Tbsp.	Carrot, potato, pumpkin, celery, and lemon		-
2nd	1st	Peanut butter *	Fruits	¼–½ cup of fruits with nut butter		2–4 Tbsp.	-
	2nd	**Watermelon**	Fruits	¼–½ cup			-
	3rd	**Sweet potato**	Fruits	¼–½ cup of fruits with nut butter	Sweet potato, pumpkin, olive oil, raw avocado, and lemon		-
	4th	**Peeled peach**	Fruits	¼–½ cup			-
	5th	**Peeled mango**	Fruits	¼–½ cup of fruits with nut butter			-
	6th	**Cauliflower**	Fruits	¼–½ cup	Carrot, sweet potato, cauliflower, and lemon	¼–½ cup	-
	7th	**Cherries**	Fruits	¼–½ cup			-
3rd	1st	Almond butter *	Fruits	¼–½ cup with nut butter			-
	2nd	**Chicken**	Fruits	¼–½ cup of well-tolerated fruits	Chicken soup (broth without flesh), carrot, potato, zucchini, celery, and lemon		-
	3rd	**Strawberries**	Fruits	¼–½ cup of fruits			-
	4th	**Peeled apricot**	Fruits	¼–½ cup of fruits with nut butter			-
	5th	**Broccoli**	Fruits	¼–½ cup of fruits	Carrot, potato, zucchini, broccoli, and lemon		-
	6th	**Plums**	Fruits and nuts	¼–½ cup of fruits with nut butter			-
	7th	**Blackberries**	Fruits	¼–½ cup of fruits			-
4th	1st	Pine nuts *	Fruits	¼–½ cup of fruits and crushed nuts	Chicken soup (broth with flesh), carrot, potato, zucchini, celery, lemon, and 1 tsp. rice		-
	2nd	**Pomegranate**	Fruits and nuts	¼–½ cup of well-tolerated fruits blended with pomegranate juice			-
	3rd	**Rice**	Fruits	¼–½ cup of fruits with nut butter			-
	4th	**Beef**	Fruits	¼–½ cup of fruits	Beef soup (broth without flesh), carrot, potato, zucchini, celery, and lemon		-
	5th	Grated walnuts *	Fruits	¼–½ cup of fruits and crushed nuts			-
	6th	**Oats**	Fruits and oats	¼–½ cup of fruits and 1 tsp. of well-boiled oat flakes			-
	7th	**Onion**	Fruits	¼–½ cup of porridge with fruits	Chicken, boiled (broth with flesh), carrot, potato, zucchini, onion, and lemon for the first 2 days, and the 3rd day, add 1–2 tsp. of bulgur		-
5th	1st	**Pineapple**	Fruits	¼–½ cup of fruits			-
	2nd	**Bulgur**	Fruits	¼–½ cup of porridge with fruits and nut butter or avocado			-
	3rd	Sunflower seeds *	Fruits	¼–½ cup of fruits and crushed nuts	Beef, boiled (broth with flesh), carrot, potato, zucchini, broccoli, and lemon		-
	4th	**Prunes**	Fruits and nuts	¼ mashed, soaked prune and ½ fruit			-
	5th	Egg yolk *	Fruits and egg	¼–½ cup of fruits with oats and egg yolk			-
	6th	**Peas**	Fruits	¼–½ cup of fruits	Mashed chicken or chopped into small pieces and boiled peas with tomato	¼–½ cup	-
	7th	**Frumenty**	Fruits	¼–½ cup of fruits and crushed nuts	Well-boiled frumenty		-
6th	1st	**Blueberries**	Fruits and nuts	¼–½ cup of fruits	Beef mashed or chopped into small pieces with 1–2 Tbsp. of mashed, boiled vegetables		-
	2nd	**Peeled kiwi, no seeds**	Fruits	¼–½ cup of fruits and crushed nuts			-
	3rd	Egg, whole *	Fruits and egg	Mixed fruits and ¼ an egg			-
	4th	**Lentils and rice**	Fruits	Fruits with ½ an egg	Rice cooked with lentils ***		-
	5th	**Pork**	Fruits	¼–½ cup of fruits with nuts	Pork meat well cooked with celery and carrots ***		-
	6th	Grated pistachio *	Fruits	¼–½ cup of fruits with nuts	Vegetable soup, in pieces, with bulgur		-
	7th	**Fresh tomato**	Fruits	¼–½ cup of fruits	Chicken in small pieces with fresh tomato and peas	¼–½ cup	-
7th	1st	**Soft cow’s milk cheese like cottage cheese**	Fruits and egg	¼–½ cup of fruits with ¼–½ an egg	Frumenty and 1 tsp. of soft cheese	¼–½ cup	May add an extra snack: e.g., 1 Tbsp. of cheese, ½ slice of bread, or ¼ toast
	2nd	**Rye bread or cracker**	Fruits and nuts	¼–½ cup of fruits with nuts	Omelet with vegetables, cheese, and ¼–½ slice of rye bread or cracker		
	3rd	**Chickpeas**	Fruits	Mix of the well-tolerated fruits	Chicken in pieces with boiled vegetables and chickpeas/orzo/okra (side differs each day)		
	4th	Orzo *	Fruits	¼–½ cup of fruits with nuts			
	5th	**Okra**	Fruits	Mix of the well-tolerated fruits			
	6th	**Octopus**	Fruits	Mix of the well-tolerated fruits	Pasta with octopus’ broth (no flesh or mashed flesh)		
	7th	**Mushrooms**	Fruits	¼–½ cup of fruits	Mushroom soup	½–1 cup	May add an extra meal and 1 snack: e.g., 1 egg 2–3 times a week either mixed with fruit or served with vegetables and avocado plus yogurt as an afternoon snack or ¼ toast
8th	1st	Fish, cod *	Fruits	Mix of the well-tolerated fruits	Fish soup (fresh cod) with vegetables and lemon		
	2nd	**Goat yogurt**	Fruits and yogurt	Mix of the well-tolerated fruits and Greek goat yogurt			
	3rd	**Tahini**	Fruits and yogurt	Mix of the well-tolerated fruits, tahini, and Greek goat yogurt		½–1 cup	
	4th	Wheat pasta *	Fruits	Mix of the well-tolerated fruits	Beef in pieces with pasta and tomato sauce		
	5th	**Figs**	Fruits	Mix of the well-tolerated fruits			
	6th	**Raisins**	Fruits	Oatmeal with yogurt and raisins			
	7th	**Soft goat cheese like Katiki Domokou, anthotyros, or manouri**	Fruits and oats	Mix of fruit with oats	Chylopites (Greek noodles with tomato sauce and 1 tsp. of katiki Domokou or other soft cheese) ***		
9th	1st	**Beans**	Fruits	Mix of the well-tolerated fruits	Water-boiled beans, 1 tsp. of cheese, and bread		
	2nd	**Lamb**	Fruits and nuts	Mix of well-tolerated fruits and nuts	Lamb with lettuce ***, cooked as fricassee		
	3rd	**Boiled spinach**	Fruits and oats	Mix of fruit with oats	Spinach with rice ***, 1 tsp. of cheese, and bread		
	4th	**Rabbit flesh**	Fruits	Mix of well-tolerated fruits	Rabbit flesh cooked with pasta ***		May add 1 extra meal and 1–2 snacks: e.g., egg as kagianas or omelet 2–3 times a week plus yogurt as an afternoon snack and barley rusk, tomato, manouri, olive oil, and oregano as dinner
	5th	Hazelnut butter *	Fruits and nuts	Mix of well-tolerated fruits and nuts			
	6th	Cow’s milk yogurt *	Fruits and Greek yogurt	Mix of the well-tolerated fruits and Greek yogurt			
	7th	Shrimp *	Fruits and oats	Mix of fruit with oats	Orzo with shrimp broth (no flesh or mashed flesh)		
10th		**Giants**	Oatmeal	Oatmeal with yogurt	Water-boiled giants with 1 tsp. of cheese and bread	½–1 cup (30 g vegetables, 30 g meat or chicken, 20–80 g of starchy food, and 1 tsp of olive oil)	
		**Corn**	Oatmeal	Mix of fruit with oats and nuts	Beef in pieces, corn, and peas with olive oil/lettuce/beet root (side differs each day)		
		**Fresh lettuce**	Oatmeal	Mix of fruit with oats			
		**Beet root**	Oatmeal	Mix of fruit with oats and nuts			
		**Yellow cheese like kasseri**	Oatmeal	Mix of fruits with oats	Traditional Greek pasta (skioufikta) with cheese and tomato sauce ***		
		**Mussels**	Oatmeal	Mix of fruit with oats and nuts	Rice with mussel broth		
		**Cooked cabbage**	Oatmeal	Mix of fruit with oats	Cabbage with rice ***, 1 tsp. of cheese, and bread		
11th		**Fresh spinach**	Oatmeal	Mix of fruit with oats and nuts	Meatballs, cooked (giouvarlakia) ***, or beef burger and salad with different fresh chopped vegetables, e.g., spinach/rocket/carrot (salad differs each day)	½–1 cup	
		**Fresh rocket**	Oatmeal	Mix of the fruits with oats			
		**Fresh carrot, chopped**	Oatmeal	Mix of fruit with oats and nuts			
		**Green beans**	Oatmeal	Mix of fruit with oats and nuts	Green beans *** cooked with tomato sauce and 1 tsp. of cheese		
		**Liver**	Oatmeal	Mix of fruit with oats and nuts	Liver with rice and fresh salad		
		**Orange**	Oatmeal	Mix of fruit with oats and nuts			
		**Fresh cabbage**	Oatmeal	Mix of fruit with oats and nuts			
12th		**Barley**	Oatmeal	Mix of fruit with oats and nuts	Zucchini with meatballs (kolokythakia gemista ***) or cabbage with meatballs (lachanontolmades ***)	½–1 cup	
		**Ariani**	Oatmeal	Mix of fruit with oats and ariani			
		**Mandarin or tangerine**	Oatmeal	Mix of fruit with oats and nuts			
		**Goat**	Oatmeal	Mix of fruit with oats and nuts	Goat flesh and boiled potatoes		
		**Eggplant**	Oatmeal	Mix of fruit with oats and nuts	Eggplant *** with 1 tsp. of cheese and bread		
		**Parsley**	Oatmeal	Mix of fruit with oats and nuts	Fish fillet boiled with parsley, olive oil, and lemon (plaki ***) with fresh or boiled salad, like greens		
		**Greens**	Oatmeal	Mix of fruit with oats and nuts			
13th		**Peppers**	Oatmeal	Mix of fruit with oats and nuts	Beef meat cooked with tomato, peppers, and pasta		2 main meals and optional 1–2 snacks
		**Anchovies or sardines**	Oatmeal	Mix of fruit with oats and nuts	Fish, anchovies or sardines, in oven with parsley and oregano	½–1 cup	
		**Artichoke**	Oatmeal	Mix of fruits with oats and nuts	Artichoke with carrots and potatoes		
		**Calamari**	Oatmeal	Mix of fruits with oats and nuts	Calamari broth with rice		
		**Quince**	Fruits and yogurt	Yogurt with boiled quince	Chicken with lemon sauce chopped into pieces with rice		
		Broad bean or fava or falafel **	Oatmeal	Mix of fruits with oats and nuts	Broad bean or fava or falafel, with 1 tsp. of cheese and bread		
		**Asparagus**	Oatmeal	Mix of fruits with oats and nuts	Eggs with asparagus		

Each new food introduced is highlighted in bold in the table. * Common allergens are noted just for the first time added. ** Broad beans should be avoided entirely by those who have glucose-6-phosphate dehydrogenase deficiency (G6PDD), also known as favism. *** Traditional dish. Tsp.: teaspoon (5 mL); Tbsp.: tablespoon (15 mL); ¼ cup: 60 mL; ½ cup: 120 mL; 1 cup: 240 mL.

**Table 3:** MedDiet foods selected during CF period according to food groups.

FRUITS	VEGETABLES	ANIMAL PRODUCTS	DAIRY PRODUCTS
Peeled pear	Boiled carrot	**Lobster**	Ariani
Peeled apple	Boiled zucchini	**Shrimp**	Yellow cheese like kasseri
Peeled banana	Boiled celery	Chicken flesh	**Soft cow’s milk cheese like cottage cheese**
Peeled avocado	Boiled potato	Beef flesh	
Peeled mango	Fresh lemon juice	Lamp flesh	
Pumpkin	Boiled pumpkin	Goat flesh	Soft goat cheese like Κatiki Domokou, anthotyros, or manouri
Melon	Boiled sweet potato	Pork flesh	
Watermelon	Boiled cauliflower	**Egg white**	
Sweet potato	Boiled broccoli	**Egg yolk**	Soft goat cheese like Katiki Domokou, anthotyros, or manouri
Peeled peach	Boiled onion	Beef liver	
Kiwi, no seeds	Cooked cabbage	Rabbit	
Berries	Boiled mushrooms	Sardines	**Cow’s milk yogurt**
Cherries	Boiled beet root	**Anchovies**	Goat yogurt
Plums	Boiled okra	**Fish, cod**	**SEEDS**
Pomegranate	Boiled spinach	Octopus	
Strawberries	Boiled corn	Mussels	Pumpkin seeds
Peeled apricot	Boiled peas	Calamari	**Sesame seeds**
Pineapple	Fresh tomato	**GRAINS**	Chia seeds
Prunes	Fresh lettuce		Sunflower seeds
Figs	Fresh spinach	Rice	**NUTS**
Raisins	Fresh rocket	**Oat**	
Orange	Fresh carrot, chopped	**Bulgur**	**Peanut butter**
Mandarin	Fresh cabbage	**Rye**	**Almond butter**
Boiled quince	Boiled greens	**Wheat**	**Grated walnuts**
**PULSES**	Boiled green beans	**Frumenty**	**Hazelnut butter**
	Boiled eggplant	**Barley**	**Grated pistachio**
Chickpeas	Fresh parsley	Amaranth	**Tahini**
Lentils	Boiled peppers	Quinoa	**Pine nuts**
Beans	Fresh peppers	Buckwheat	**ADDED OIL**
Giants	Boiled artichoke	Pasta	
Broad bean or fava	Boiled asparagus	Orzo	Extra virgin olive oil

Common allergens are in bold in the table [20].

**Table 4:** RF recommendations for caregivers [49].

Parameters	Guidance for Caregivers
Feeding environment	Be present and pleasant during the meal (e.g., verbalization, eye-to-eye contact, and not forcing the child to eat)
Quality	Insist repeated exposure to accept new foods
Quantity	Encourage self-feeding and self-regulation in infants and toddlers
Hunger and satiety cues	Identify and respond in an emotionally supportive and predictable way to hunger and satiety cues, recognizing changing cues as the child develops, and differentiating hunger from other issues that may cause an infant or young child to fuss or cry
Soothing	Do not use food to calm a child when she/he is not hungry
Introduction of complementary foods	Introduce complementary foods in a timely way, considering child developmental readiness
Flavor preferences	Offer a diverse diet with repeated exposures to healthy foods/beverages and avoid offering ultra-processed foods and sugar-sweetened beverages
Food consistency	Offer foods with appropriately evolving consistency as the child develops
Portion sizes	Offer foods and beverages in the child’s own plate or bowl and with other eating utensils (e.g., spoon) that are appropriate for the developmental stage and nutritional needs of the child
Caregiver feeding styles	Be patient and do not pressure the child to eat or finish food or limit food intake
Nurturing feeding environment	Provide loving and stimulating verbalizations to the child
Eat as a family	Be a role model preparing and consuming healthy foods/beverages in a clean and pleasant nurturing environment
Avoid distractions during feeding	Screens or cell phones during feeding should being avoided
Daily routines	Established, well-structured daily routines for eating, sleeping, playing, and bathing helps

**Table 5:** Proper time for adding new foods.

Food Group	Proper Time for Introducing CF
Fruits and vegetables [29]	From the 4th month of life in small quantities
Gluten-free grains [29]	From the 4th month of life in small quantities
Meat (red and white) [19]	Until the end of the 7th month of life
Yogurt and cheese [19]	From the 6th month of life in small quantities
Foods containing gluten [29]	No earlier than the 4th and no later than the 7th month of life
Eggs [29,50]	From the 4th to the 6th month of life
Peanuts [26,44,50]	From the 4th to the 11th month of life
Fish [32]	Before the 12th month of life
Legumes [19]	Before the 12th month of life
Fresh milk [19]	After the 12th month of life
Common allergens [32,44]	From the 4th and before the 12th month of life

**Table 6:** Foods and beverages to be avoided or limited to 12 months of age [1,19,52].

Foods and Beverages to be Avoided
Foods in forms that may cause choking
Foods high in sugar and trans fats
Tea and herbal infusions
Table salt and salty products
Sugar-sweetened beverages
Non-sugar sweeteners
Honey
Whole nuts and fish with bones
Fresh milk
Processed meat
Commercial fruit juices
**Foods and Beverages to be Limited**
Consumption of 100% fruit juice
Rice products
